# Medical Schools

**Published:** 1852-09

**Authors:** 


*Medical Schools.* — A new medical school has been organized in Cincinnati. Prof. Mussey, lately connected with the Ohio Med. College, has become one of the faculty of the new school. The chair of anatomy and of the theory and practice of medicine, in the Medical College of Ohio, made vacant by the resignation of Drs. Mussey and Bell, have been filled by the election of Drs. Drake and Cobb, lately connected with the Med. Department of the University of Louisville. The vacant chairs in the latter institution have been filled by the election of Drs. Palmer and Flint, of the Medical Department of the University of Buffalo.

